# Analysis of geometrical tomographic parameters of furcation lesions in periodontitis patients

**DOI:** 10.1016/j.heliyon.2021.e06119

**Published:** 2021-01-30

**Authors:** Bianca Costa Gonçalves, Andre Luiz Ferreira Costa, Raquel Correa, Naira Maria Rebelatto Bechara Andere, Celso Massahiro Ogawa, Mauro Pedrine Santamaria, Sérgio Lucio Pereira de Castro Lopes

**Affiliations:** a Department of Diagnosis and Surgery, São José dos Campos School of Dentistry, São Paulo State University (UNESP), São José dos Campos, SP, Brazil; b Postgraduate Program in Dentistry, Cruzeiro do Sul University (UNICSUL), São Paulo, SP, Brazil

**Keywords:** Cone beam computed tomography, Digital volume tomography, Furcation defect, Furcation involvement, Periodontal regeneration, Periodontal disease

## Abstract

**Objectives:**

This study was aimed to investigate the relationship between geometric parameters of furcation lesions - maximum area of lesion opening (MALO), angle formed between the roots (ABR), lesion volume (LV) and presence and height of infra-osseous defects (IOD) - and the success of therapy with enamel matrix derivative proteins (EMD) in patients with grade C periodontitis, using cone-beam computed tomography (CBCT).

**Methods:**

The study consisted of two groups of patients with grade C periodontitis: control (surgery) (n = 17) and test (surgery + EMD) (n = 17). Images parameters on CBCT were recorded using OnDemand3D and ITK-SNAP software.

**Results:**

Pearson's correlation coefficient demonstrated that only IOD was statistically significant in the probing depth PD (*P* = 0.01), with a moderate positive correlation (R = 0.59). MALO was found to be statistically significant (*P* = 0.03) in the test group (surgery + EMD), with moderate negative correlation (R = -0.5).

**Conclusion:**

The presence of infra-osseous defects and height were relevant in relation to the success of the type of treatment addressed in this study.

## 1 Introduction

The current treatment for grade C periodontitis involves initial, basic and, if necessary, surgical interventions. The initial intervention consists of oral hygiene instructions and oral environment adequacy. Mechanical procedures are part of the basic intervention in which open flap debridement (OFD) is considered the gold standard for treating periodontal diseases. For treatment of grade C periodontitis, however, single mechanical intervention may not reduce periodontal pathogens in affected sites as the disease may persist [1].

Therefore, several authors have searched for therapeutic alternatives to improve the treatment and prognosis of patients with grade C periodontitis [1, 2, 3], in which regenerative therapy is a plausible option with significant advantages for the patient. The main purpose of regenerative periodontal therapy is to restore both anatomy and function of the damaged periodontium, which includes new bone formation to the base of the defect, cementum and periodontal ligament. Several regenerative strategies have been evaluated in comparison to OFD, including root surface conditioning, bone grafts and bone substitute materials, guided tissue regeneration, enamel matrix proteins, growth/differentiation factors, combined therapies and, more recently, tissue-engineering approaches [4].

Among the aforementioned regenerative approaches, there is a technique using enamel matrix derivative proteins (EMD), in which amelogenins are the main component. EMD has brought significant improvements in the regenerative treatment of periodontal defects caused by periodontal disease. These proteins have the ability to promote periodontal ligament regeneration, in addition to increasing the formation of mineralized matrix and to releasing growth factors. Studies have shown that EMD can also reduce the local pathogenic flora, thus creating a more favorable environment for periodontal regeneration [5].

Diagnostic imaging is a valuable tool for complementing clinical examinations. Conventional radiographic methods, including periapical and panoramic radiographs, have been frequently used to estimate the degree of furcation lesions. However, these methods are inherently limited as they provide only two-dimensional images, and consequently, overlapping structures and varying degrees of distortion may occur. Three-dimensional images obtained with cone beam computed tomography (CBCT) allow these structures to be reliably evaluated and, therefore, this technique should be considered for the study of periodontal diseases [6, 7].

CBCT is currently considered a reliable imaging technique for diagnosis and treatment of furcation lesions, including infra-osseous defects and other periodontal problems [8]. Research has addressed the use of CBCT in the diagnosis of periodontal diseases, particularly furcation lesions [6, 9, 10].

Although there are studies addressing the importance of CBCT in the diagnosis of furcation lesions, there are a few ones specifically addressing their geometrical parameters.

In this context, based on the promising role of EMD in the periodontal regeneration process and on the fact that CBCT is a high-capacity imaging technique for analysing periodontal lesions, in addition to providing different three-dimensional information on the periodontal lesion and involved tooth, this study aimed to investigate the relationship between tomographic parameters of furcation lesions in upper molars and the success of EMD therapy in patients with grade C periodontitis.

## 2 Patients and methods

### 2.1 Patient selection

The sample selection was conducted at the Periodontics Clinic of the São José dos Campos School of Dentistry and Institute of Science and Technology of the São Paulo State University (ICT-UNESP) and the subjects for this study were a subset of a previously research of aggressive periodontitis.

The sample consisted of 34 patients diagnosed with grade C periodontitis who met pre-established criteria for inclusion.

Grade C periodontitis corresponds to one in which there is a rapid rate of progression, that is, loss greater than or equal to 2.0 mm in more than five years, and destruction exceeding the expectation for biofilm deposits with specific clinical patterns suggestive of periods of rapid progression and/or early-onset disease (e.g. molar/incisor pattern, lack of expected response to standard bacterial control therapies).

This study was approved by the local human research ethics committee according to protocol number #1.734.857 and was conducted in accordance with the Helsinki Declaration of 1975, as revised in 2000. All patients provided written informed consent to participate.

The inclusion criteria were as follows:

I) Aged between 18 and 35 years old.
II) Presence of grade C periodontitis [11] in at least six sites containing periodontal pocket and insertion loss greater than 5.0 mm with bleeding on probing and two more pockets with insertion loss greater than 6.0mm also with bleeding on probing, being at least three pockets located in single-rooted teeth and not adjacent.
III) Presence of at least 20 teeth.
IV) Good systemic health.
V) Signing an informed consent form agreeing to participate in the study, after explanation of the risks and benefits.

The Exclusion criteria were as follows:

I) Presence of systemic problems (e.g. diabetes, cardiovascular alterations such as atherosclerosis, blood dyscrasias, immunodeficiency, among others) contra-indicating the periodontal procedure;
II) Previous periodontal treatment in the last 12 months;
III) Previous use of antibiotics and anti-inflammatory drugs in the last six months;
IV) Being smoker, pregnant or lactating;
V) Chronic use of medications affecting the response of periodontal tissues.

### 2.2 Tomographic evaluation of furcation lesions according to the parameters

Patients were submitted to further scan by using an I-CAT Next Generation unit (Imaging Science International, Hattfield, PA, USA) operating at a 0.125-mm voxel size, 40-second image acquisition, 37.07 mA, 120 kVp, voxel size of 0.20 mm, exposure time of 26.9 s and field of view (FOV) of 8.0 × 8.0 mm.

The images acquired were converted into DICOM (Digital Imaging and Medical Communications) format for use with OnDemand3D (Cybermed Inc., Tustin, CA, USA) and ITK-SNAP 1.4.1 (University of North Carolina, Chapel Hill, NC, USA) software. All images were measured by one calibrated investigator who was trained by a radiologist with experience of 6 years in CBCT images, in addition to being previously calibrated and unaware of the treatment received by the patients. Examiner calibration was conducted as follows: the investigator measured the MALO, ABR, VDL and IOD of ten patients twice within a 24-hour interval. Subsequently, the measurements were submitted to intraclass correlation test and the examiner was considered calibrated as the agreement was above 90%.

### 2.3 Maximum area of lesion opening (MALO)

To determine the MALO, the images were converted into DICOM format for use with OnDemand 3D software. Initially, the occlusal plane of each patient was previously corrected by using the axis-cutting tool on the images so that it was always parallel to the horizontal plane, thus establishing a standardized process.

**Figure 1:**
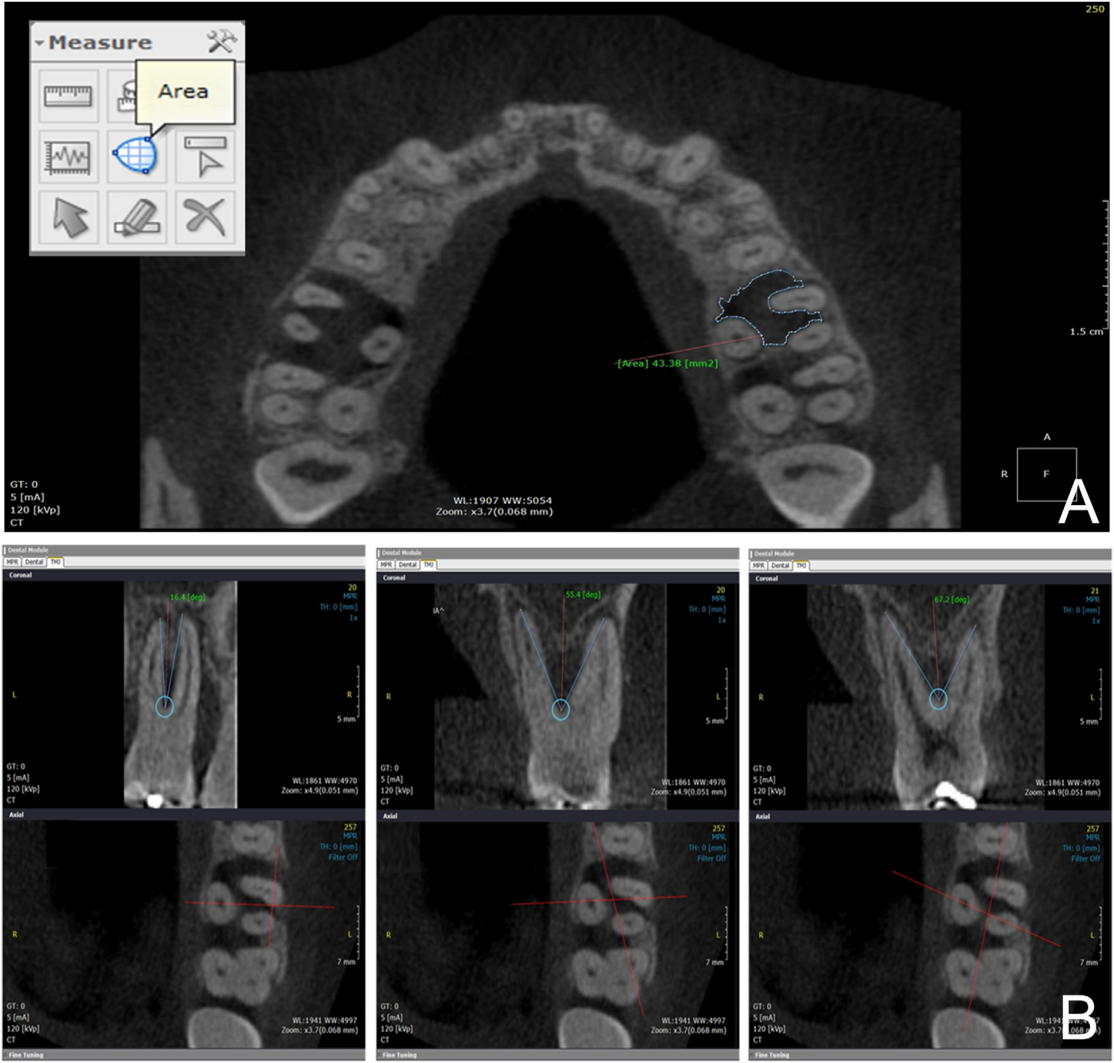
A: axial section showing the delimitation of the lesion area in mm^2^. B: Measurements of the angles between the roots, two by two: mesio and buccal; mesio-buccal and palatal; disto-buccal and palatal.

This software also has an area tool by which each section of the structure can be delimited (i.e. segmentation process), providing a value in mm^2^. To this end, the multiplanar reconstruction mode (MPR) was used in which axial slices are viewed in the occlusal-apical direction, allowing the furcation lesion to be observed in the tooth (Figure 1A). After reconstructing all the slices, the furcation lesion was segmented by using the Area tool in which the slices could be visually broader, resulting in values corresponding to the areas. Next, these areas were tabulated and then their largest value corresponding to the MALO was determined.

### 2.4 Angle formed between the roots (ABR)

In order to obtain an individual angular measurement between the roots of the three maxillary molars under study, the CBCT DICOM images were exported to the OnDemand3D software. The axial section was selected by using the TMJ tab in the axial window, thus making it possible to visualize the middle thirds of the three roots. Next, by using the Curve tool, a segment line was drawn, two by two, around the roots of the tooth studied. This segment line resulted in a perpendicular cut allowing individualized view of the roots in their full extent. These cuts were scanned to determine which one could allow the best view of the roots. The Angle tool was then used so that the vertex of this angle coincided with the furcation region of the roots and the angle's sides coincided with the long axis of the roots, ending at their apex. Next, the ABR was determined in degrees (Figure 1B). This process was repeated for every two roots in order to obtain individual ABRs between mesial and distobuccal roots, between mesiobuccal and palatal roots and between palatal and distobuccal roots.

### 2.5 Lesion volume (LV)

**Figure 2:**
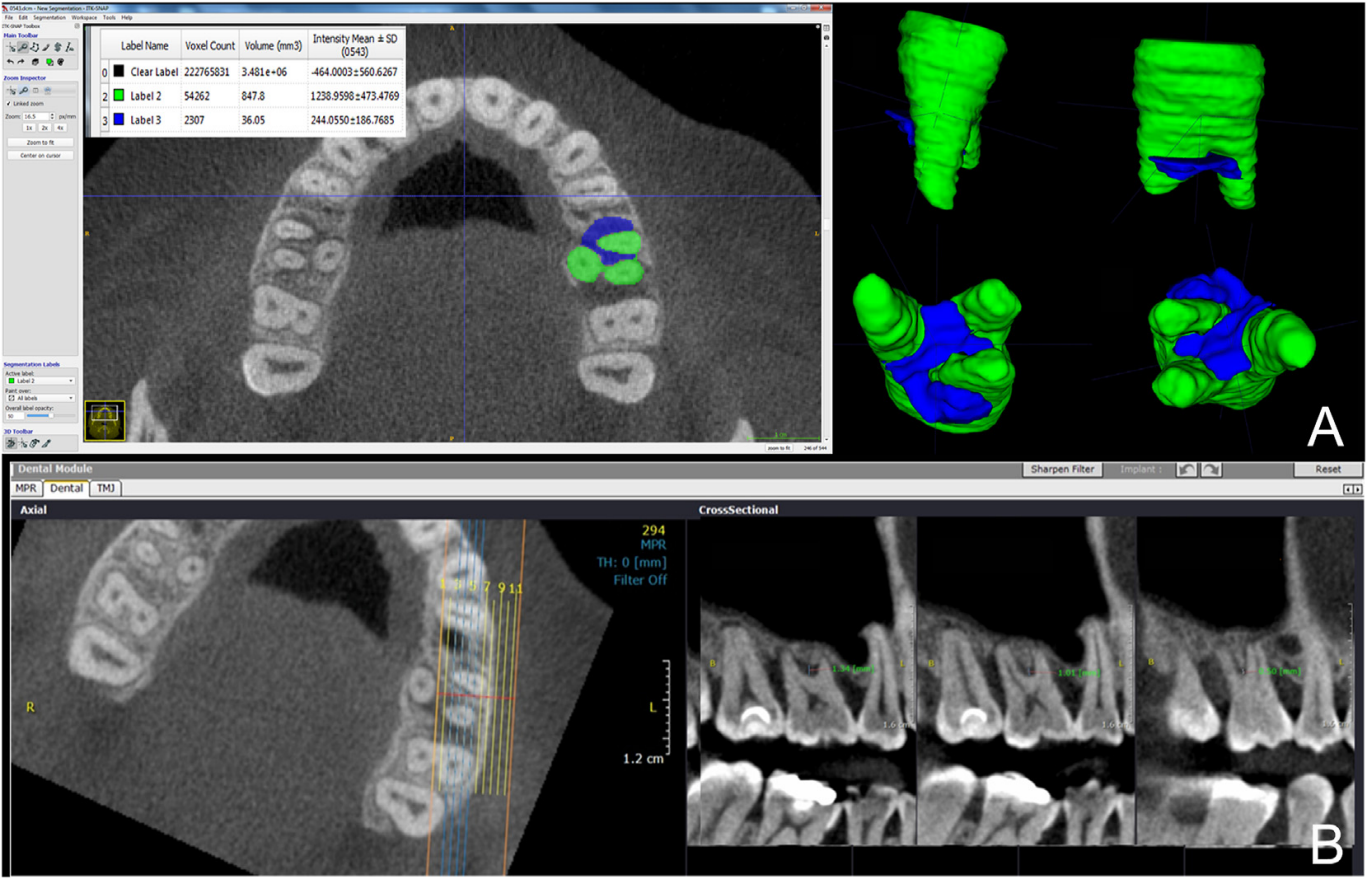
A: Axial section showing the segmentation of the lesion (blue) and tooth (green), in mm^3^ and reconstruction model. B: Axial section showing the semi-straight line equidistant to the buccal roots (i.e. MB and DB). The cross-section window is vertically measured with the ruler tool from the furcation to the bone level.

LV was obtained by using the ITK-SNAP 3.4.0 software (University of North Carolina, Chapel Hill, NC, USA), allowing the axial sections of the images to be visualized, that is, the region(s) of interest of the tooth with furcation lesion. In order to obtain the volume, manual segmentation was conducted by using the Polygon tool, in which the lesion was delimited in the axial sections to generate its individual area. At the end of this process, that is, after all the lesions had their areas segmented in the occlusal-apical direction, the volume of the lesion was calculated based on the segmented areas (Figure 2A).

### 2.6 Presence and height of infra-osseous defects (IOD)

For this, the images were evaluated by using the OnDemand3D software, more specifically the Dental module. Initially, by means of the Axis-cutting tool, the long axis of the tooth was adjusted so that it remained perpendicular to the horizontal plane.

Next, in the axial section, the elements in question were located in the middle thirds of the three roots (i.e. mesiobuccal - MB, distobuccal – DB, and palatal - P). With the Arc tool, a semi-straight line was drawn equidistant to the buccal roots (i.e. MB and DB) and perpendicular to an imaginary line joining them. This semi-straight line generated perpendicular buccopalatal cuts automatically, which were displayed in the cross-section window. This enabled to gradually follow the bone levels from the entrance of the furcation between these roots to their central region, which were vertically measured with the Ruler tool from the furcation to the bone level, thus providing values of the matching bones. Next, the same procedure was repeated considering the MB and P roots as well as DP and P roots, two by two, thus determining the presence of infra-osseous defect considering each face of the furcation (Figure 2B).

Three heights were progressively measured when bone defect was observed between the roots, that is, in the palatal, intermediate and buccal regions of the furcation, corresponding to the same infra-osseous defects. After the measurements, root defects were averaged for each tooth studied.

**Figure 3:**
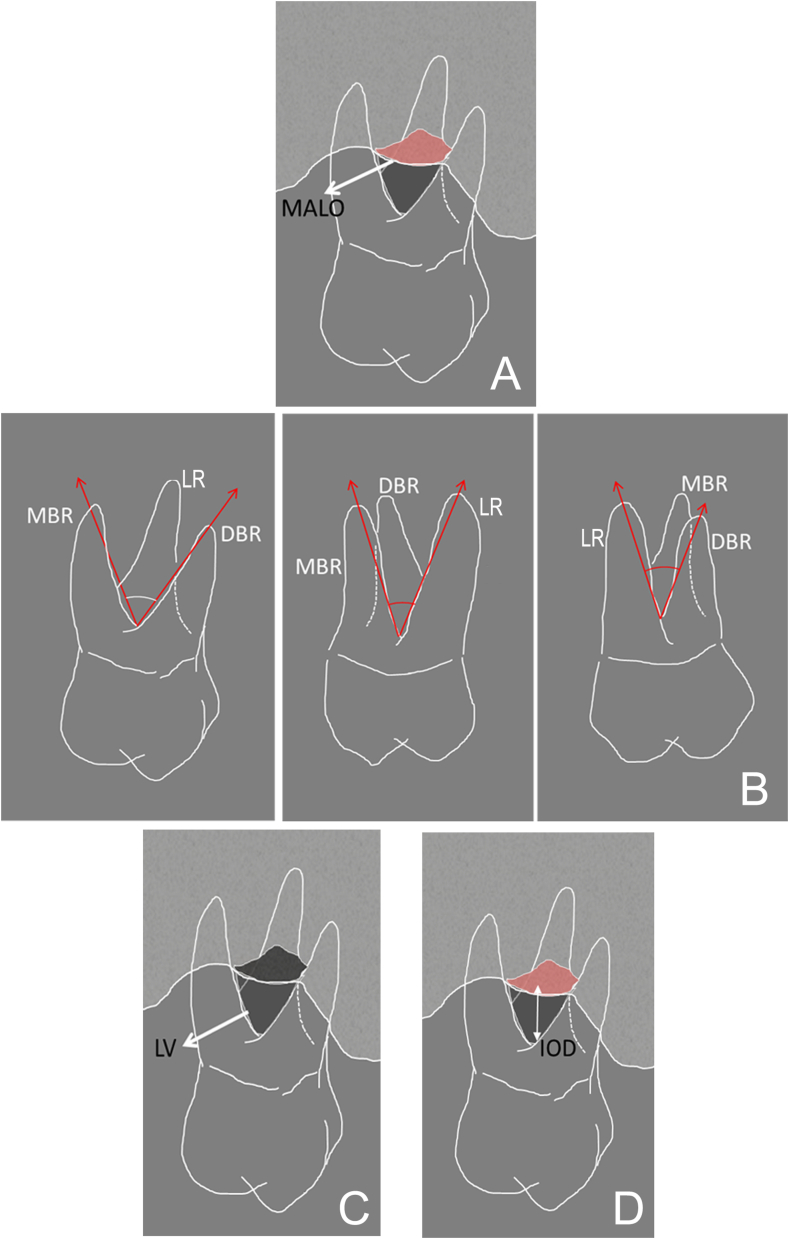
Schematic view of the tomographic parameters: A) MALO (maximum area of lesion opening); B) MBR (mesial angle formed between the roots, LR (lingual angle formed between the roots), DBR (distal angle formed between the roots); C) LV (lesion volume); D) IOD (presence and height of infra-osseous defects).

The tomographic parameters are shown in Figure 3.

### 2.7 Periodontal therapy

This was a prospective, randomized, controlled, clinic triple-blinded study. All patients underwent an initial treatment aimed at adequacy of the oral environment, in which they received information about their periodontal status before being instructed about oral hygiene and undergoing supragingival calculus scraping, prophylaxis and coronary polishing.

All patients underwent an initial treatment of grade C periodontitis, which consisted of oral hygiene counseling, prophylaxis, adequacy of the oral environment, supragingival scraping and ultrasound debridement of the whole mouth, as well as information about their periodontal status. Even after periodontal debridement, the patients received oral hygiene instruction so that they could achieve a plaque index <20%. During these sessions, supragingival scraping was also conducted to maintain optimal oral hygiene.

After re-assessment, proximal sites of maxillary molars with grade 2 furcation involvement, probing depth (PD) of 5 mm or greater, vertical and horizontal insertion loss ≥5 mm, and presence of bleeding were distributed according to the quadrant to be treated as follows:

I) Test group (OFD + EMD): Periodontal scraping and root planing + EMD application (Emdogain, Straumann).
II) Control group (OFD): Periodontal scraping and root planing.

Surgical treatment was conducted by a single operator as follows: antisepsis of the facial region was performed with 0.2% chlorhexidine digluconate; sterile eye drops for perioral skin disinfection; and 10 mL of 0.12% chlorhexidine digluconate as mouth-rinse solution (Periogard®, Colgate Palmolive Ltda, Osasco, SP, Brazil) for 1 min for intraoral disinfection. After the preoperative procedures, local anesthesia with 2% mepivacaine and epinephrine at 1:100,000 (DFL - Rio de Janeiro, RJ, Brazil) was injected in the patient by infiltration technique. Next, OFD was performed according the modified Kirkland surgery flap technique (Kirland, 1931). With a 15-cm blade, intrasulcular incisions were made to the bottom pocket in the buccal, lingual and interproximal areas, extending in the mesial and distal directions. The gingival tissue was detached from the underlying bone tissue to expose the root surfaces, which were then scraped and flattened. Visible granulation tissue and calculus were manually removed with the aid of curettes (Gracey, Hu-Friedy) and ultrasonic device (Cavitron, Dentsply, Tulsa, OK, USA) was used for scraping. Diagnosis of grade II furcation was confirmed by using Nabers probe (Hu-Friedy). At this point there is a treatment randomization.

The test group received application of EMD as specified by the manufacturer (Emdogain, Straumann). After scraping, the site was abundantly irrigated with saline solution. After hemostasis, the root surfaces were kept dry and then EMD gel was applied.

All clinical measurements were conducted by a single examiner (NCCS), who was previously calibrated and unaware of the treatment given to the patients. The examiner calibration was done as follows: The examiner measured the PD and clinical insertion level of 15 patients twice within 24 h. Next, the measurements were submitted to intraclass correlation test and the examiner was considered calibrated as a 90% agreement was reached. Evaluations were conducted prior to treatment (baseline) and after six months. The following parameters were evaluated: plate index, gingival index, PD – measured from the bottom of the pocket to the gingival margin by using a North Carolina periodontal probe (UNC-Hu-Friedy), and horizontal clinical insertion level (HCAL) – horizontal distance between clinical insertion into the furcation and the base of the stent, measured by using a modified Nabers probe. All measurements were expressed in millimeters to assess the horizontal component of the defect (Suh et al. 2002). Furcation defects were classified according to Hamp et al. [12] by using a Nabers probe (Hu-Friedy).

### 2.8 Statistical analysis

Statistical analysis was performed by using independent t-test or Mann-Whitney's test and Chi square test for demographic characteristics of the patients. A combined result was defined as “Delta” (Δ): final values minus initial values for PPD and HCAL. The comparisons between groups were performed by using two-way ANOVA and Pearson's correlation test.

All statistical analyses were performed by using the software SAS System for Windows, version 9.4. (SAS Institute Inc, Cary, NC, USA) at a significance level of 5%.

## 3 Results

Analysis of the demographic data was performed, which is shown in Table 1. One can observe that there were no statistically significant differences regarding age and gender (*P* > 0.05).

**Table 1:** Demographic characteristics of patients (n = 34).

	Surgery (n = 17)	Surgery + EMD (n = 17)	*p* value
Age (years)	30,27 ± 5,77	32,94 ± 5,26	0,1^a^
No of teeth	26,94 ± 1,47	27,29 ± 1,31	0,5^b^
Gender	2/15	2/15	1^c^

a Test T.

b Mann-Whitney Rank Sum Test.

c Chi Square.

Clinical results at the baseline and six months after the treatment are shown in Table 2. With regard to the probing depth (PD) and horizontal clinical attachment level (HCAL), there were no statistically significant differences at the baseline and six months after the treatment (*P* > 0.05). Additionally, it can be emphasized that there were no significant variations in PPD or HCAL at the baseline and after six months in both test and control groups.

**Table 2:** Clinical results of baseline and 6 months furcation sites.

Clinical parameters	Time	Surgery	Surgery + EMD	P value Intergroup
PD (mm)	Baseline	5,6 ± 1,15Aa	5,35 ± 1,0Aa	0,7
	6 months	5,05 ± 1,27Ba	4,85 ± 1,34Ba	0,4
	ΔPD	0,57 ± 1,1a	0,51 ± 1,2a	0,3∗
HCAL (mm)	Baseline	6,7 ± 1,5Aa	6,5 ± 1,72Aa	0,6
	6 months	6,35 ± 1,17Aa	6,08 ± 1,4Ba	0,2
	ΔHCAL	0,35 ± 1,12a	0,42 ± 1,23a	0,6∗

PD, probing depth; HCAL, horizontal clinical attachment level; Distinct uppercase letters: statistically significant difference (p < 0.05) in intragroup (vertical) comparison. Two Way Repeated Measures ANOVA. Distinct lower-case letters: statistically significant difference (p < 0.05) in intergroup comparison (horizontal). Two Way Repeated Measures ANOVA. ∗p value, T test.

The Pearson's correlation test was used to assess the possible presence of correlations between tomographic parameters (volume, angle, defect and area) and clinical parameters (reduction of PD and increase of HCAL) in both groups (surgery and surgery + EMD). The results are shown in Table 3.

**Table 3:** Correlation analysis of dependent variables, ΔPD e ΔHCAL.

ΔPD	Surgery (n = 17)		Surgery + EMD (n = 17)	
	0,57 ± 1,1a		0,51 ± 1,2a	
	R	p value	R	p value
Volume	0,34	0,1	0,22	0,3
Angle	-0,3	0,2	0,36	0,1
Defect	0,59	0,01	0,01	0,9
Area	0,45	0,06	-0,36	0,1
ΔNICH	0,35 ± 1,12a		0,42 ± 1,23a	

	R	p value	R	p value

Volume	-0,39	0,1	-0,09	0,7
Angle	-0,17	0,5	0,29	0,2
Defect	0,12	0,6	0,17	0,5
Area	0,05	0,8	-0,5	0,03

PD, probing depth; HCAL, horizontal clinical attachment level; Values de R (Pearson Coefficient) e p (p value)– Pearson correlation test.

Considering the reduction of probing depth (ΔPD) in the control group, it is possible to observe in Table 3 that there was a statistically significant difference (*P* = 0.01) in the height of the furcation defect, with a moderately positive correlation (R = 0.59), indicating that the reduction in PPD was greater in cases where the defect already existed and presented higher values. With regard to the maximum area of lesion opening (MALO), it was observed that although there was no statistically significant difference (*P* = 0.06), there was a tendency for this to occur. Therefore, Pearson's correlation coefficient was used and showed that this relationship tends to be moderately positive (R = 0.45), that is, the greater the value of the lesion area, the greater the reduction in probing depth (ΔPD). In the test group (surgery + EMD), no statistically significant differences were observed for all tomographic parameters in relation to the reduction in probing depth (ΔPD).

With regard to the horizontal clinical attachment level (ΔHCAL), only the test group showed a statistically significant difference in relation to the maximum area of lesion opening (*P* = 0.03), with moderately negative correlation (R = -0.5), that is, the larger the maximum area of lesion opening, the lower the gain in horizontal clinical attachment level (ΔHCAL).

Table 4 shows the correlation analysis of the dependent variables ΔPPD and ΔHCAL when data from both groups were analysed together (overall data). One can observe that when both groups are analysed together, there were statistically significant differences in lesion volume (*P* = 0.05) and defect height (*P* = 0.04) in relation to the reduction in probing depth (ΔPD), with Pearson's correlation coefficient showing a moderately positive correlation (R = 0.3 and 0.34, respectively). This indicates that in larger volume lesions there was an increased tendency of reduced probing depth. Similarly, this behavior was also observed in the defect height when the groups were analysed separately (Table 3). It is important to emphasize that, overall, no statistically significant differences were observed in the gain in horizontal clinical attachment level (ΔHCAL) for all parameters.

**Table 4:** Correlation analysis of dependent variables. ΔPD and ΔHCAL. when data from both groups were analyzed together.

ΔPD	Overall Data (n = 34)	
	R	p value
Volume	0.3	0.05
Angle	0.01	0.9
Defect	0.34	0.04
Area	0.11	0.5
ΔHCAL	R	p value
Volume	-0.24	0.1
Angle	0.07	0.7
Defect	0.03	0.8
Area	0.25	0.1

PD, probing depth; HCAL, horizontal clinical attachment level; Values de R (Pearson Coefficient) e p (p value)– Pearson correlation test.

## 4 Discussion

The anatomical characteristics of the dental elements, especially maxillary molars, have some peculiarities such as presence of three roots, root divergence and furcation. In fact, the presence of periodontal disease may cause difficulties in the clinical management and unpredictability in response to regenerative periodontal therapy, which emphasizes the importance of a study taking into account all anatomical parameters of the teeth involved [5]. In this context, this study aimed to evaluate the role of these anatomical characteristics in the response to periodontal treatment in patients with periodontal disease (i.e. grade C periodontitis, which may belong to stages 3 or 4).

Some studies found in the literature evaluated the influence of anatomical characteristics on the success of regenerative periodontal therapy, in which root divergence, lesion anatomy and presence of furcation lesions could change the regenerative response [13, 14]. However, there are few data on the proximal furcation involvement in maxillary molars, especially in patients with grade C periodontitis.

In the present study, it was observed that there was a significant reduction of probing depth in the control group when infra-osseous defect in the furcation was present (as can be seen in Table 3). On the other hand, this behavior was not observed in the test group. Analysis of the primary data showed that the average defect height was higher in the control group, which may explain our results as the literature shows that deep defects were improved in the clinical attachment level after periodontal therapy. In Table 4, one can observe that the results further support this hypothesis [15, 16, 17].

When the correlation between maximum area of lesion opening and clinical data was calculated in the control group (surgery), it was observed that there was a statistical behavior indicating that the larger this area, the greater the reduction in the probing depth (Table 3). A possible explanation for this finding could be based on the fact that a larger tooth surface (i.e. maximum area) would provide a larger site for tissue reorganization with recruitment of repairing cells, thus decreasing the PPD in this specific group [18].

In the test group (surgery + EMD), the results were controversial regarding the correlation of tomographic parameters with the reduction of probing depth, since no statistical differences were observed in any of the variables. Some anatomical factors could have contributed to this result, such as lower frequency of infra-osseous defects in the test group compared to the control group. In addition, minor defects were more present in the test group than in the control group, a finding observed with analysis of primary data on an individual basis. Additionally, studies [19, 20] show that EMD has better results when used in class III furcation lesions. On the other hand, our study investigated class II furcation lesions. Volume of the lesion is another anatomical factor possibly influencing the results as it was considerably higher in the test group (surgery + EMD). It is known that large furcation lesions commonly have a closer proximity to the oral environment, a fact which might explain the difficult tissue regeneration. This, in turn, would significantly increase the clinical parameters due to the greater contact with salivary flow and its resident pathogenic bacteria. Nibali and colleagues [21] showed that molars with furcation involvement near the gingival margin were associated with greater probing depth, mobility and radiographic vertical bone loss, meaning that it is possible to infer these results from analyses using CBCT. This theory may also explain the fact that, in the test group, there was a lower gain in HCAL when the maximum area of opening lesion was larger (as it can be seen in Table 3).

It is important to emphasize that the literature shows that EMD has no mechanical properties to assist proving space and stabilization of the clot (6), requiring a larger structure around the lesion. Therefore, the larger lesion volume observed in the test group may have impaired the periodontal regeneration process, although there are several studies in the literature showing its predictability [5, 20, 22].

Some studies have been conducted by using this material in combination with other techniques [23, 24] to explain the EMD's behavior regarding its need of a steady environment for adaptation. Murphyu and Gunsolley [25] found that membrane application to infra-osseous defect may result in a gain of more than 1.0mm in the clinical attachment level compared to the open flap debridement. Other study showed evidence suggesting that the combined use of EMD and grafts was effective to treat infra-osseous defects [26].

Among the limitations of this study, one can cite the patient's difficulty in maintaining proper oral hygiene and plaque control in the surgical sites, which may either directly or indirectly influence the results. Casarin et al. [5] used the same procedure during periodontal maintenance sessions, in which patients with furcation lesions underwent regenerative periodontal therapy and had plaque in the surgical sites. In addition, studies suggest that the microbiota in furcation lesions is much more heterogeneous than that in the interproximal sites [27]. In fact, the furcation anatomy may vary significantly between the individuals, which can determine the microbial diversity [4, 27, 28]. Therefore, it is reasonable that molars with furcation involvement are at higher risk of lesions, even in patients undergoing supportive periodontal care [21].

When the two groups were analysed together (overall data), it was observed that the larger the lesion volume, the deeper the probing depth (as shown in Table 4). This finding seems to be controversial regarding the results observed in the test group (surgery + EMD) when analysed separately. It is possible that a larger volume cannot correspond to a maximum area of opening lesions. Moreover, the depth of large defects, as seen before, is inversely applied to this result when the clinical parameters are reduced (PD reduction and HCAL gain). Statistical analysis of the two groups (Table 4) showed that the test group (surgery + EMD) had smaller values of maximum lesion and defect height, as mentioned above, with antagonistic influence on the overall result.

In this analysis of the clinical data (Table 2), one can observe that there was no statistical improvement between the groups in the parameters probing depth and clinical attachment level. One fact that may have contributed to this result is that the evaluation was conducted six months after the surgery, and some studies show clinical improvement over longer post-operative periods [5, 15, 29]. This fact constitutes another limitation of our methodology.

Within the limitations of this study, we did not analyse vertical components. Further studies evaluating these parameters are warranted.

Despite the parameters analysed in both groups, there was no relationship between the angle formed by the roots and clinical parameters. Some studies [14, 30] show that root divergence can influence the response of periodontal therapy, but this result was due to the conventional radiographic evaluation. In this pioneering study, the root angles were objectively assessed by taking individual measurements and using CBCT. Therefore, our results may be relevant regarding this variable. Overall, the results indicate that the presence of infra-osseous defects are an important factor to be evaluated in the periodontal treatment, regardless of the regenerative techniques used, showing its association with reduced probing depth.

One should consider that the present study is the first to correlate anatomical parameters, use of CBCT and clinical success of the regenerative technique in patients with grade C periodontitis. However, further studies with larger samples and longer postoperative periods should be conducted to confirm our results, including tomographic approaches enabling a better spatial acquisition (i.e. lower voxel values) and use of post-processing image filters improving evaluation of the measurement parameters.

## 5 Conclusion

The results of this study show that, among the tomographic parameters analysed, a greater height of IOD and a larger area of MALO suggest that there is a greater reduction in the depth of PD, especially in the control group. In the study group, a greater MALO tended to cause a greater gain in clinical insertion (ΔHCAL). Additionally, joint analysis showed that the volume of the largest lesion would be related to a greater reduction in the probing depth.

## Declarations

### Author contribution statement

B. Gonçalves: Conceived and designed the experiments; Performed the experiments; Wrote the paper.

A. Costa: Conceived and designed the experiments; Analyzed and interpreted the data; Wrote the paper.

R. Correa: Performed the experiments; Analyzed and interpreted the data.

N. Andere and M. Santamaria: Performed the experiments; Analyzed and interpreted the data; Contributed reagents, materials, analysis tools or data.

C. Ogawa: Analyzed and interpreted the data; Contributed reagents, materials, analysis tools or data.

S. de Castro Lopes: Conceived and designed the experiments; Performed the experiments; Analyzed and interpreted the data; Wrote the paper.

### Funding statement

This work was supported by 10.13039/501100001807FAPESP (São Paulo Research Foundation) (grants no. 2016/15143-0, 2017/05101-0, 2018/17850-0 and 2018/11997-0).

### Data availability statement

Data will be made available on request.

### Declaration of interests statement

The authors declare no conflict of interest.

### Additional information

No additional information is available for this paper.
